# Comparative analysis of different survey methods for monitoring fish assemblages in coastal habitats

**DOI:** 10.7717/peerj.1832

**Published:** 2016-03-21

**Authors:** Duncan G.L. Baker, Tyler D. Eddy, Reba McIver, Allison L. Schmidt, Marie-Hélène Thériault, Monica Boudreau, Simon C. Courtenay, Heike K. Lotze

**Affiliations:** 1 Department of Biology, Dalhousie University, Halifax, Nova Scotia, Canada; 2 Fisheries and Oceans, Gulf Fisheries Centre, Moncton, New Brunswick, Canada; 3 University of Waterloo, Canadian Rivers Institute, Waterloo, Ontario, Canada

**Keywords:** Fish assemblage, Environmental monitoring, Beach seine, Coastal zone management, Underwater visual census (UVC), Survey selection

## Abstract

Coastal ecosystems are among the most productive yet increasingly threatened marine ecosystems worldwide. Particularly vegetated habitats, such as eelgrass (*Zostera marina*) beds, play important roles in providing key spawning, nursery and foraging habitats for a wide range of fauna. To properly assess changes in coastal ecosystems and manage these critical habitats, it is essential to develop sound monitoring programs for foundation species and associated assemblages. Several survey methods exist, thus understanding how different methods perform is important for survey selection. We compared two common methods for surveying macrofaunal assemblages: beach seine netting and underwater visual census (UVC). We also tested whether assemblages in shallow nearshore habitats commonly sampled by beach seines are similar to those of nearby eelgrass beds often sampled by UVC. Among five estuaries along the Southern Gulf of St. Lawrence, Canada, our results suggest that the two survey methods yield comparable results for species richness, diversity and evenness, yet beach seines yield significantly higher abundance and different species composition. However, sampling nearshore assemblages does not represent those in eelgrass beds despite considerable overlap and close proximity. These results have important implications for how and where macrofaunal assemblages are monitored in coastal ecosystems. Ideally, multiple survey methods and locations should be combined to complement each other in assessing the entire assemblage and full range of changes in coastal ecosystems, thereby better informing coastal zone management.

## Introduction

Field surveys are a common first step for many fundamental and applied ecological studies, and provide an essential foundation for environmental management (Connolly, 1994; Mapstone & Ayling, 1998; Hernandez et al., 2006; França, Costa & Cabral, 2009; França, Costa & Cabral, 2011). As anthropogenic impacts on natural ecosystems increase, mapping and monitoring are increasingly valued as a means to quantify changes in the environment. Thus, the ability to complete accurate, rapid and cost-effective species surveys has become an important conservation and management tool (Hughes, 1996; Schmitt, Sluka & Sullivan-Sealey, 2002; Langlois et al., 2010; Murphy & Jenkins, 2010).

For most natural communities, a variety of different survey methods exist; yet resulting estimates of species abundance and diversity can vary with the survey method selected, influencing both the precision and accuracy of the data (Connolly, 1994; Mapstone & Ayling, 1998; Côté & Perrow, 2006). Thus, data collected using different methods may not be directly comparable due to survey biases. Given that survey results may be used for conservation and management decisions, it is therefore important to evaluate how different survey methods perform, and whether they can replace or complement each other (Murphy & Jenkins, 2010; Ward-Paige, Flemming & Lotze, 2010).

In estuarine and coastal ecosystems, monitoring mobile macrofaunal assemblages, particularly fish assemblages, requires underwater survey techniques. Commonly used methods monitoring mobile macrofaunal assemblages in estuarine and coastal ecosystems include beach seine netting, underwater visual census (UVC), baited remote underwater video (BRUV), diver-operated stereo video, as well as more destructive methods such as dredging/trawling, poisoning, and suction sampling (Harmelin-Vivien & Francour, 1992; Côté & Perrow, 2006; Murphy & Jenkins, 2010; Holmes et al., 2013). Both BRUV and stereo video have the advantage of reduced diver error of size and abundance estimation, yet BRUV has been criticized for preferentially surveying greater numbers of large predatory fish species, without seeing comparable changes in smaller herbivorous species (Watson et al., 2005; Langlois et al., 2010). Additionally, both BRUV and stereo video have the downside of increased survey equipment costs and data analysis time due to large amount of footage (Willis, Millar & Babcock, 2000; Langlois et al., 2010).

In comparison, the beach seine is a simple, rapid and cost-effective way to sample a relatively large, clearly defined area and allowing for live collection of individuals (Piercer, Rasmussen & Leggett, 1990). However, it has been criticized for its variable capture efficiencies; as the net may snag or roll allowing for escapees, small species may avoid capture in depressions in the sea bed, faster swimming fish may vacate the area while the seine is cast, and it can cause mortality of captured individuals, such as vulnerable juvenile fish (Piercer, Rasmussen & Leggett, 1990; Connolly, 1994; Weldon et al., 2004). Also, dragging the net over the seafloor can cause some disturbance, and catching large schools of fish that cannot be processed quickly may lead to some mortality (authors’ personal observation). Similar to the beach seine, when accessible from shore and a boat is not required, visual surveys are simple and quick, quantitative and repeatable, can be accurately done by relatively novice divers, and have minimal impact on the environment (Harvey, Fletcher & Shortis, 2001; Schmitt, Sluka & Sullivan-Sealey, 2002; Edgar, Barrett & Morton, 2004; Ward-Paige & Lotze, 2011). However, visual surveys also have biases; the air-water interface in the diver mask leads to size magnification and changes in depth perception (though methods exist to standardize size estimates), divers often under- or overestimate the area sampled, and low visibility in turbid waters may lead to reduced observations (Ross, Franklin & Weitman, 1969; Harvey, Fletcher & Shortis, 2001; Harvey et al., 2004). Visual surveys can underestimate the abundance of the most common species, and species behaviour may affect the results as some species are attracted to, while others may swim away from the diver (Brock, 1982; Chapman & Atkinson, 1986; Dickens et al., 2011). Visual surveys also often miss cryptic species; however, interference visual census (IVC) is a technique which dismantles habitat to survey cryptic species, and can sample these species better than nets. However, this requires sampling twice the amount of transects and has a greater impact on the habitat (Beldade & Gonçalves, 2007). Finally, since UVC requires instantaneous identification of individuals in an open environment, it may result in species misidentification. Despite the widespread use of both beach seines and UVC for monitoring coastal ecosystems around the world, there are few studies that have evaluated the comparability of these survey methods in different regions (Franco et al., 2012).

Another important question in the design of monitoring programs is where to perform surveys. Most coastal ecosystems consist of a diverse set of habitats with various bottom types, foundation species, depths, abiotic conditions and anthropogenic impacts, and associated macrofaunal assemblages likely differ among different habitat types and human impacts (Heck, Hays & Orth, 2003; Schmidt et al., 2011; Schein et al., 2012; Holmes et al., 2013). Thus, depending on the goal of the monitoring program, it is important to decide which survey methods to use, and where to perform them in order to gain the most meaningful results. Broader assessments of fish assemblages in different habitats within and among estuaries have been performed in Europe, albeit using more destructive trawl surveys (França, Costa & Cabral, 2009; França, Costa & Cabral, 2011; Selleslagh et al., 2009).

**Figure 1:**
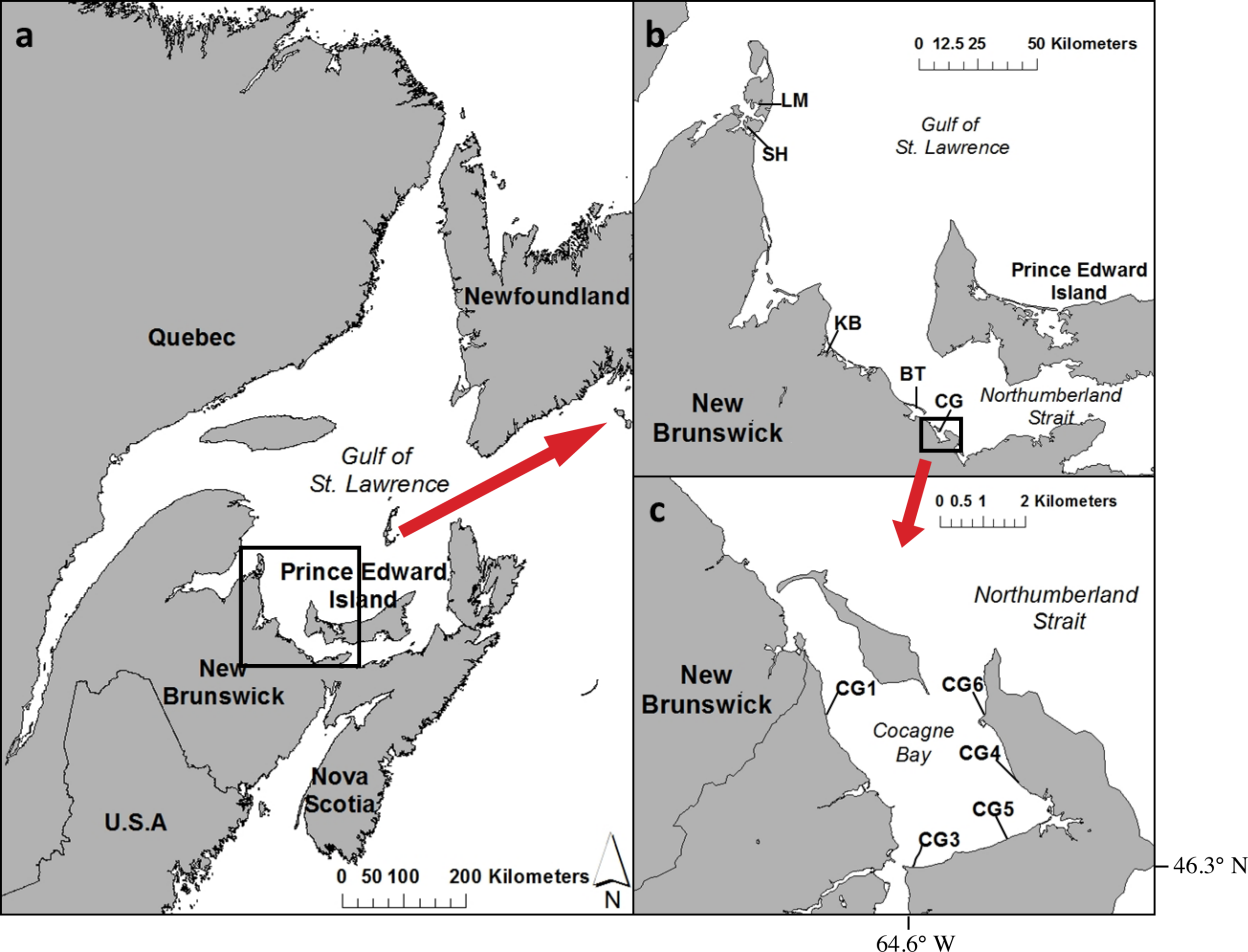
Map of (A) Atlantic Canada with the southern Gulf of St. Lawrence and Northumberland Strait region of New Brunswick, (B) a close-up of the five estuaries sampled: LM, Laméque; SH, Shippagan; KB, Kouchibouguac; BT, Bouctouche; CG, Cocagne, and (C) map of Cocagne Bay with long-term CAMP study sites CG1 to CG6 (ESRI, 2011; Government of Canada GeoGratis, 2013).

Here, we use a case study from Atlantic Canada, where both beach seine and UVC surveys have been used to monitor coastal fish assemblages at a regional scale, to assess differences in survey methods and survey location. Since 2003, the Department of Fisheries and Oceans (DFO) Community Aquatic Monitoring Program (CAMP) has been using beach seine surveys in multiple estuaries along the coast of the southern Gulf of St. Lawrence (Fig. 1A) to assess changes in coastal fish assemblages (Weldon et al., 2004). Data collection is frequently completed by trained local community organizations, maximizing spatial and temporal sampling. At the same time, researchers from Dalhousie University have been using UVC surveys to assess macrofaunal assemblages in eelgrass beds among multiple estuaries in the same region (Lotze et al., 2003; Coll et al., 2011; Schmidt, 2012; Schmidt et al., 2012). Many estuaries in the southern Gulf of St. Lawrence are dominated by eelgrass (*Zostera marina*), which has been designated an ecologically significant species (ESS, DFO, 2009) because it provides important ecosystem services such as spawning, nursery and foraging habitat for a wide range of associated species (Vandermeulen, 2005; Vandermeulen, 2009; DFO, 2009). However, eelgrass habitats are threatened by a number of human impacts such as nutrient loading and aquaculture operations (Lotze et al., 2003; Schein et al., 2012; Schmidt et al., 2012; Skinner, Courtenay & McKindsey, 2013). Currently, efforts are underway to develop a monitoring framework for the region led by the Northumberland Strait Environmental Monitoring Partnership (NorSt–EMP; www.cwn-rce.ca/). One objective of this partnership is to determine how and where to best monitor coastal fish assemblages and eelgrass beds.

The objectives of our study were to compare estuarine mobile macrofaunal assemblages sampled by two commonly employed survey methods; beach seine and UVC, as well as mobile macrofaunal assemblages found in two common habitats; nearshore and eelgrass. The results have important implications for how and where to best sample coastal fish assemblages to assess changes in the structure and functioning of coastal ecosystems. Our results can be used for the design of NorSt–EMP as well as other national or international monitoring programs already using or adopting these common monitoring methods.

## Materials and Methods

### Study sites

From August 6th–13th 2013, mobile macrofaunal assemblages were sampled among five estuaries in the southern Gulf of St. Lawrence, Canada to assess among estuary variability (Figs. 1A and 1B, Table S1). Estuaries were selected to be spread out along the Northumberland coast, and within each estuary, one site was randomly selected from a pool of four possible sites, located in the mid-estuary, about halfway between the river’s main freshwater inflow and the estuary’s main outflow into the Gulf of St. Lawrence (Lotze et al., 2003). Within one of these five estuaries—Cocagne Bay—additional sampling of another four sites was undertaken to assess within-estuary variability to compare to among-estuary variability (Fig. 1C, Table S1). Sampling was undertaken during the summer due to logistical constraints, but also because it has been shown to be the season with the greatest diversity in mobile macroinvertebrates, although spring and fall are important seasons for fish spawning and recruitment (Schmidt & Scheibling, 2007). All study sites were located in sheltered areas and shallow waters (<2 m deep) with large (>50 m wide) continuous eelgrass beds close to shore. Water temperatures ranged from 19.6 to 22.4 °C, salinities from 22.6 to 27.2 PSU, and dissolved oxygen content from 5.2 to 13.8 mg/L (Table S1).

All procedures performed in this study involving animals were in accordance with the ethical standards of the institutions at which the studies were conducted. The Dalhousie University Committee on Laboratory Animals approval number is: #11–103. This field research was conducted under the scientific licence to the Department of Fisheries and Oceans, # SG-RHQ-13-089.

### Sampling design

Our sampling aims were twofold: (i) to compare beach seine and visual census (UVC) surveys in nearshore habitats within one estuary (Cocagne Bay) and among five estuaries; (ii) to compare nearshore and eelgrass habitats among five estuaries (Table S1). All sampling was completed between 9:00 and 16:00, and followed the design of existing survey protocols by CAMP and Dalhousie (Weldon et al., 2004; Schmidt et al., 2011; Schmidt, 2012).

**Figure 2:**
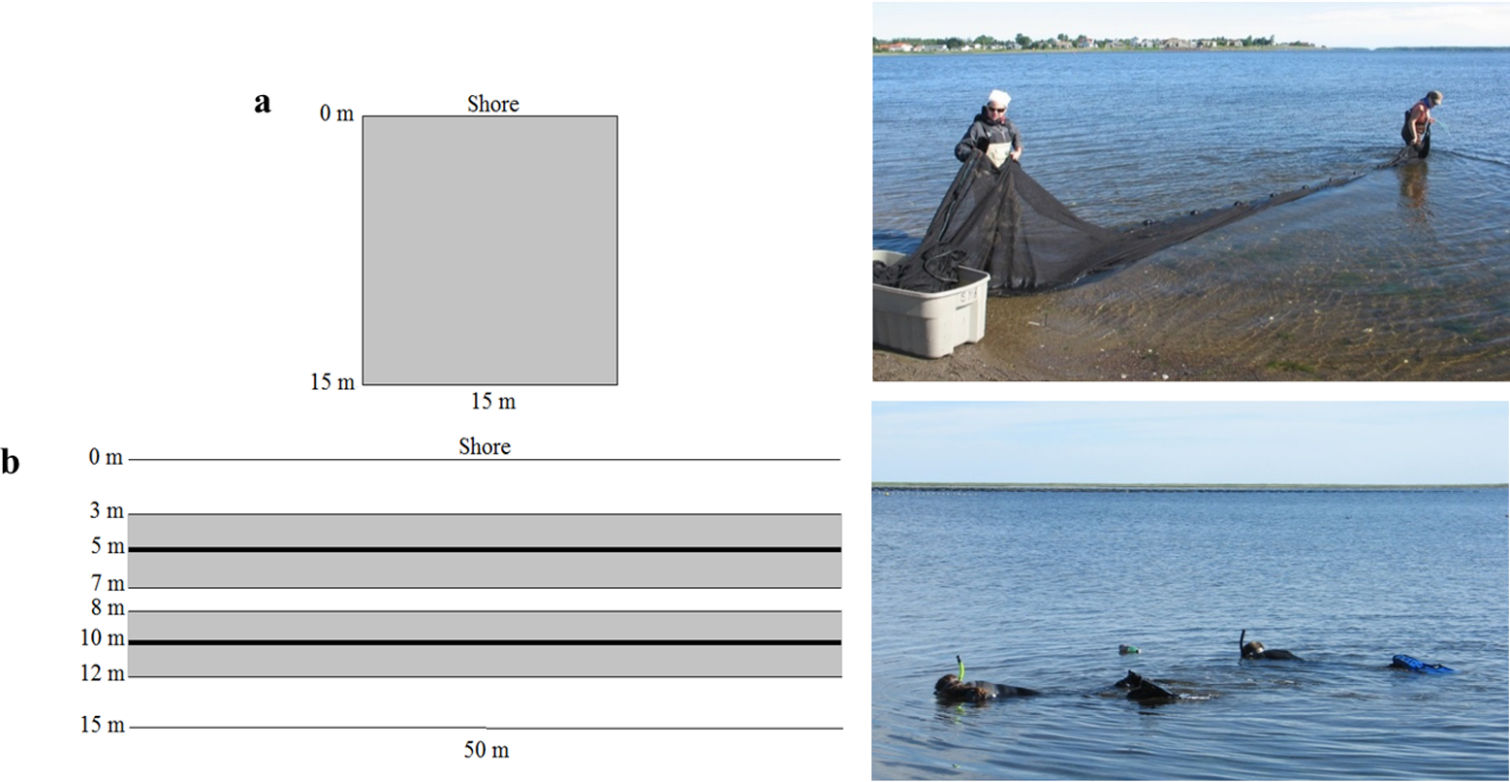
Sampling layout and photograph of (A) beach seine and (B) underwater visual census (UVC) surveys in nearshore shallow water with marked distances from shore and survey lengths. Shaded areas represent those sampled by the given method; a total of 225 m^2^ in the beach seine and 200 m^2^ in each visual survey. UVC transect lines at 5 m and 10 m from shore are bolded (Photo credit: Heike Lotze).

For the survey method comparison, at each of the nine study sites (Figs. 1B and 1C), two 50 m transects were placed parallel to shore (at 5 m and 10 m distance from shore) and left undisturbed for a minimum of 20 min prior to UVC nearshore sampling in order to allow for any disturbance caused by placing the transects to subside. UVC nearshore sampling was undertaken at high tide due to the shallow depths surveyed (0.75–1.5 m in depth) and to survey mobile species that use the intertidal zone during the high tide (Fig. 2B). During high tide, two divers swam parallel, each on a separate transect. All mobile macrofauna (fish and crustaceans such as crabs) within 2 m of either side of the transect line were counted, sized and identified to species level if possible. Hence, the total area sampled per transect was 200 m^2^ (Fig. 2B). Diver estimates of fish length were compared to species length at maturity to determine life stages and separate juveniles from adults (Table S2). UVC surveys were undertaken with a minimum of two metres of visibility to ensure that results were not being driven by ability to observe species. We recognize that the use of stereo video would aid in species identification and size estimation, however we did not have access to this equipment at the time of the surveys, and previous surveys had not employed it.

A minimum of 20 min after the UVC was completed to attempt to minimize any changes in assemblage due to time of day, tide or change in currents, a beach seine survey was performed in the same area using a 30 m long, 2 m deep seine net with 6 mm mesh size throughout (Fig. 2A). One end of the net was anchored on the beach and the other end was walked perpendicular to the shore out to 15 m, then parallel to the shore for 15 m, and then back to the beach, covering a total area of 225 m^2^ (Fig. 2A). Once cast, the net was pulled to shore from both ends forcing organisms into the central bag, from where they were transferred into a live holding tank. All individuals were counted and identified to species levels if possible, and had their life stage recorded as either young of the year or adult based on size, before being released alive back into the water. In instances where the volume of individuals collected rendered counting time harmful to the captured organisms, rare individuals were counted first, while common species abundances were estimated by subsamples of one dip net. The number typically caught in one dip net was multiplied by the number of dip nets necessary to empty the whole holding tank.

For the habitat comparison between nearshore shallow waters and eelgrass beds among five estuaries, UVC was performed using the same methods as above, but in the centre of an eelgrass bed in close proximity (50–100 m) to, but slightly deeper (0.75–1.50 m deep) than the nearshore (0–0.75 m deep) survey (Fig. 1B, Table S1). Water depths at the eelgrass sites necessitated the use of SCUBA equipment when sampling, whereas the nearshore shallow site sampling was completed using snorkelling gear. In both cases it was possible to observe species found on the benthos as well as in the water column.

### Data analysis

In order to evaluate within and among estuary variation explained by survey method and habitat, we employed a multivariate approach with the factors: survey method (beach seine vs. UVC) and habitat (nearshore vs. eelgrass). Additionally, for the within-estuary analysis, we included the factor site, and habitat was nested within site. We tested for variation in the abundance, richness, diversity, evenness, and composition of the fish and mobile macrofauna assemblage explained by method at five sites within one estuary (Cocagne) and among five estuaries. We also investigated the variation in abundance, richness, diversity, evenness, and composition of the fish and mobile macrofauna assemblage explained by habitat (nearshore vs. eelgrass) among five estuaries. Non-parametric permutational analyses of variance (PERMANOVA) were performed using PRIMER with PERMANOVA+ software (Anderson, Gorley & Clark, 2008).

To examine differences in the species composition, we used non-metric multidimensional scaling (MDS) and multivariate PERMANOVA, based on a zero-adjusted Bray-Curtis similarity matrix of log transformed species abundance data (Clarke & Gorley, 2008a). Log (*x* +*1*) transformation allows both rare and dominant species to more evenly influence the sample similarity metric, while not completely disregarding species abundances (Clarke & Gorley, 2008b). Additionally, we tested for homogeneity in dispersion (PERMDISP) of data that might be explaining observed variation (Anderson, Gorley & Clark, 2008). Similarity percentage (SIMPER) and principle coordinates (PCO) analyses were used to identify which species contributed the most to the differences in species composition (there was very high agreement between the tests, so only SIMPER results are presented), and separate univariate PERMANOVA was used to test for significant differences in the abundance of these species between survey methods and habitats (Clarke & Gorley, 2008a; Anderson, Gorley & Clark, 2008).

To investigate differences between survey methods (beach seine vs. UVC), the two shallow water visual transects (200 m^2^ each) were pooled, creating one sample to be compared with the beach seine survey (totalling 225 m^2^). Data were analyzed among the five estuaries to test for differences among estuaries (*n* = 5), and within one estuary only (Cocagne Bay) to assess within-estuary variability (*n* = 5). For the habitat comparison among the five estuaries, we kept the two transects per habitat as separate samples. We assessed differences in univariate summary measures including total abundance (per m^2^), species richness, diversity (Shannon index) and evenness (Pielou index). Univariate PERMANOVA on the zero-adjusted Bray-Curtis similarity matrix for total abundance and a Euclidean distance matrix for richness, diversity and evenness was used to test if these summary measures were different between survey methods and habitats (Clarke & Gorley, 2008a).

## Results

### Comparison of survey methods

**Figure 3:**
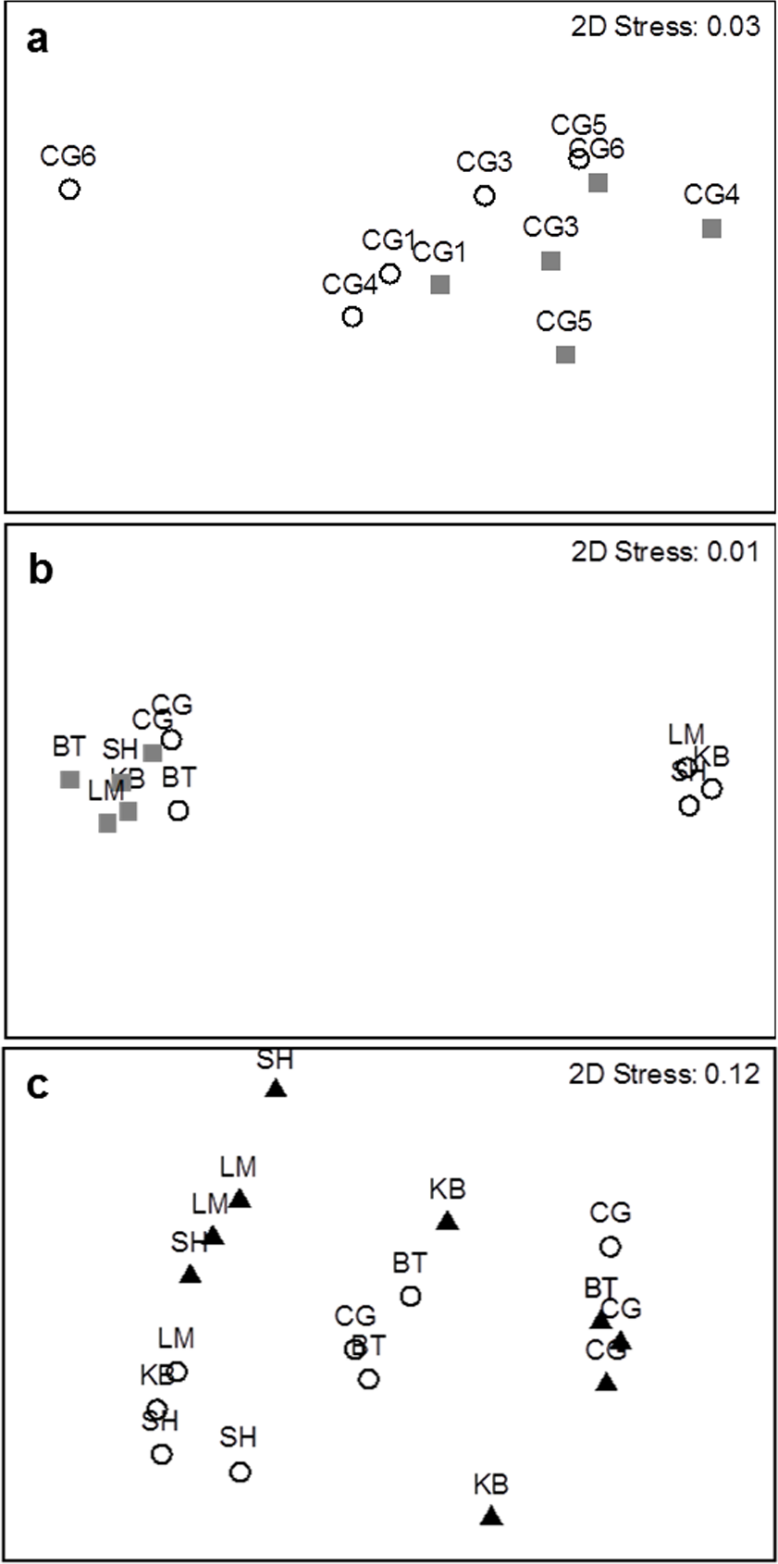
MDS plots showing groupings of species composition by study site between (A) different survey methods (open circles = visual survey, grey squares = beach seine) within an estuary (*n* = 5), (B) among estuaries (*n* = 5), and (C) different habitat types (open circles = shallow nearshore, black triangles = eelgrass beds, *n* = 5 with 2 transects each). In (C) three outliers (one KB and one LM in shallow, and one BT in eelgrass) were removed. See Fig. 1 for study site codes.

MDS plots show a clear distinction in species composition between the two survey methods within Cocagne Bay and among the five estuaries (Fig. 3A and 3B), while PERMANOVA identified significant differences in species composition among all sites and among estuaries, but not within Cocagne Bay (Table 1). Interestingly, two separate groupings for UVC appear among estuaries (Fig. 3B), where Cocagne and Bouctouche had been sampled before and the other three estuaries after a strong rainfall, which reduced visibility and resulted in lower abundance and species richness counts in Kouchibouguac, Shippagan and Lamèque (Fig. S1).

**Figure 4:**
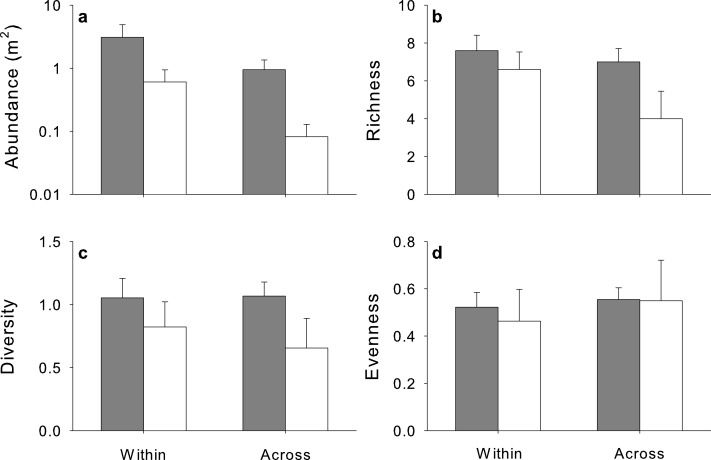
Mean (+SE) (A) abundance, (B) species richness, (C) diversity and (D) evenness sampled by visual surveys (light grey) and beach seines (dark grey) within one estuary, Cocagne Bay (*n* = 5), across estuaries (*n* = 5).

Total macrofaunal abundance varied strongly among study sites and between survey methods (Fig. S1A). Abundances were higher with the beach seine (Fig. 4) among all sites and among estuaries, but not within Cocagne Bay (Table 1). These results remained even after one outlier with very high abundance was removed (beach seine at Cocagne 4, Fig. S1A). Species richness, diversity and evenness showed strong variation among study sites (Figs. S1B–S1D) but did not differ among areas in any of the study designs (Fig. 4 and Table 1).

**Table 1:** PERMANOVA results for differences in assemblage structure between survey methods (beach seine vs. visual census) within an estuary (*n* = 5), among estuaries (*n* = 5), and among all sites (*n* = 9). All results are from univariate PERMANOVA, except for species composition, which is from a multivariate analysis. Significant *p*-values (<0.05) are indicated in bold.

	Abundance		Richness		Diversity		Evenness		Composition	
	*Pseudo-F*	*P*	*Pseudo-F*	*P*	*Pseudo-F*	*P*	*Pseudo-F*	*P*	*Pseudo-F*	*P*
Within	1.28	0.29	0.79	0.44	1.34	0.28	0.90	0.35	1.35	0.23
Among	5.58	**0.0086**	3.90	0.13	1.39	0.11	1.06	0.31	4.23	**0.0078**

**Figure 5:**
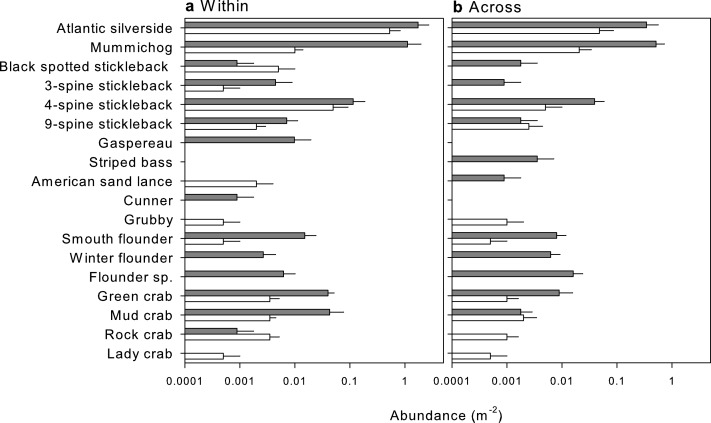
Mean abundance (+SE) of species identified by beach seines (dark grey) and visual surveys (white) in nearshore habitats (A) within one estuary, Cocagne Bay (*n* = 5) and (B) across estuaries (*n* = 5).

In total, 18 species of mobile macrofauna were encountered in the two survey methods among all study sites (Fig. 5 and Table S3), with 13 species observed in the UVC and 16 species with the beach seine. Species that were only observed in the UVC included the grubby and lady crab, while black-spotted and 9-spine stickleback and rock and mud crabs were observed in higher abundance in the visual census (Fig. 5). In turn, species only observed with the beach seine included gaspereau, striped bass, cunner, winter flounder and other flounder, while Atlantic silverside, mummichog, three-spine stickleback, four-spine stickleback, smooth flounder and green crab were observed in higher abundance with the beach seine (Table S3).

**Table 2:** Results of SIMPER analysis identifying the contribution of individual species to differences in species composition between survey methods (beach seine vs. visual census) within one estuary and Among all five estuaries, as well as univariate PERMANOVA results for differences in each species’ abundance between methods, except for juvenile flounder sp. which was only detected in the beach seine. The total contribution explained by the species listed is 92.67% for within and 91.93% for among estuaries. Significant *p*-values (<0.05) are indicated in bold.

SIMPER species	Contribution (%)	*Pseudo-F*	*P*
**Within estuary**			
Atlantic silverside	47.9	0.64	0.63
Mummichog	30.4	4.12	**0.01**
4-spine stickleback	10.0	0.45	0.97
Green crab	4.4	23.14	**0.03**
**Among estuaries**			
Mummichog	50.6	8.52	**0.02**
Atlantic silverside	30.8	0.63	0.63
4-spine stickleback	7.3	0.27	1.00
Flounder species	3.3	NA	NA

SIMPER analysis identified that most of the variation between survey methods was driven by two of the most abundant species, Atlantic silverside and mummichog, both within and among estuaries (Table 2). Both species had higher average abundance with the beach seine (Fig. 5), yet this was only statistically significant for mummichog (Table 2). Four-spine stickleback also showed higher average abundance, not significantly different than the beach seine, but had a much lower contribution to the difference in species composition between survey methods both within (10.0%) and among (7.3%) estuaries (Table 2). Two other species contributed less than 5% to the differences in species composition: green crab was significantly more abundant with the beach seine within Cocagne Bay, and juvenile flounder sp. was only sampled with the beach seine among estuaries (Table 2).

### Comparison of habitat differences

**Figure 6:**
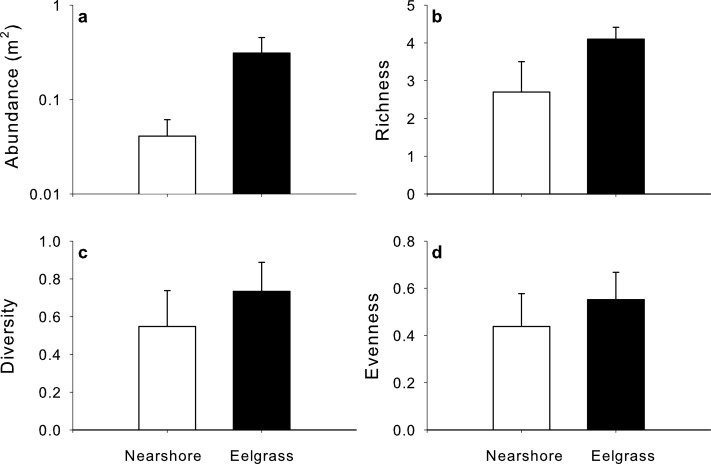
Mean (+SE) (A) abundance, (B) species richness, (C) diversity and (D) evenness sampled by UVC surveys in nearshore shallow (light grey) and nearby UVC surveys in eelgrass beds (dark grey) among five estuaries (*n* = 5).

Total abundance, richness, diversity and evenness of mobile macrofauna varied among estuaries, yet were on average higher in eelgrass than nearshore habitats (Fig. 6, Fig. S2). The factor habitat was statistically significant for all except for evenness, and generally explained a larger proportion of the variation in the model than the residuals (Table 3), while site alone explained less variation and was not significant.

**Figure 7:**
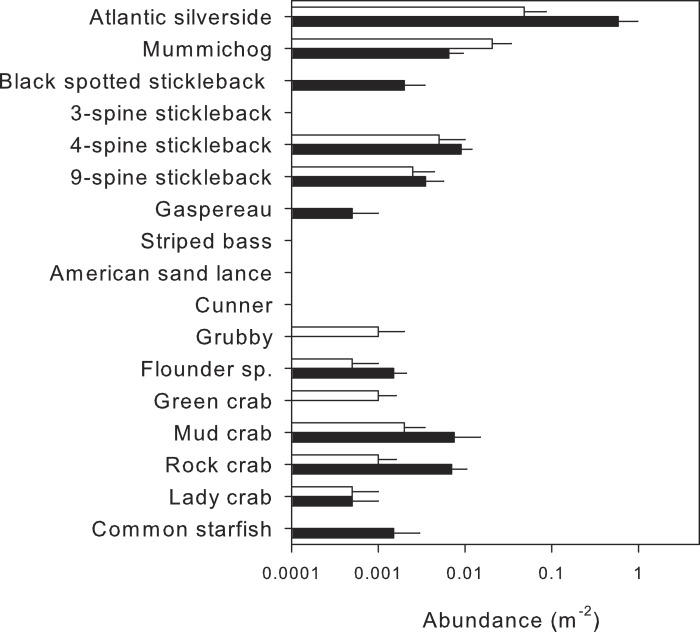
Mean abundance (+SE, *n* = 5) of species identified by visual surveys in shallow nearshore (white) and nearby eelgrass (black) habitats.

In total, 14 mobile macrofaunal species were encountered in the two habitats (Fig. 7, Table S4), of which 10 were detected in nearshore and 12 in eelgrass surveys. Two species, green crab and grubby, were only detected nearshore, and mummichog was more abundant nearshore. In turn, four species were only found in eelgrass, including gaspereau, black-spotted stickleback and common starfish; however, both gaspereau and black-spotted stickleback had previously been observed in the nearshore during the methods comparison (Fig. 5).

Despite some overlap, the MDS plot (Fig. 3C) and multivariate PERMANOVA (Table 3) confirmed that species composition differed significantly between the two habitats, and habitat explained 40.2% of the variation. SIMPER analysis indicated that Atlantic silverside explained 57.8% of the variation in species composition, while mummichog (12.1%), rock Crab (11.5%), 4-spine stickleback (7.19%) and black spotted stickleback explained much less (3.25%). All these species had higher abundance in eelgrass, except for mummichog (Fig. 7); however, these differences were only statistically significant for Atlantic silverside, where habitat explained 47.0% of the variation in abundance (Table 4). For the other species, most of the variance was unexplained.

**Table 3:** PERMANOVA results and percent variation explained for differences in assemblage structure with site and habitat (nearshore vs. eelgrass) nested in site (S). All are univariate PERMANOVA analyses, except species composition, which is a multivariate analysis. Significant *p*-values (<0.05) are indicated in bold.

	Site			Habitat (S)			Residuals
	*Pseudo-F*	*P*	% variation	*Pseudo-F*	*P*	% variation	% variation
Abundance	1.89	0.13	27.4	3.43	**0.002**	34.4	29.1
Richness	1.35	0.31	9.6	3.82	**0.019**	19.5	15.2
Diversity	0.89	0.50	0	3.95	**0.024**	28.3	21.2
Evenness	1.66	0.29	17.2	2.63	0.094	22.1	22.3
Composition	1.14	0.30	13.9	2.55	**0.001**	40.2	45.5

**Table 4:** Results of SIMPER analysis identifying the contribution of individual species to differences in species composition between habitats (nearshore vs. eelgrass), and univariate PERMANOVA results for each species’ abundance for the factors site and habitat with percent of variation explained. The total contribution explained by the species listed is 91.79%. Significant *p*-values (<0.05) are indicated in bold; NA indicates that no tests could be performed because of zero occurrences.

SIMPER species	Contribution (%)	Site			Habitat (S)			Residuals
		*Pseudo-F*	*P*	% variation	*Pseudo-F*	*P*	% variation	% variation
Atlantic silverside	57.78	0.57	0.80	0	4.12	**0.04**	47.0	37.6
Mummichog	12.07	0.87	0.56	0	1.38	0.31	19.4	39.2
Rock crab	11.51	0.54	0.82	0	NA	NA	NA	50.7
4-Spine stickleback	7.19	7.03	0.14	29.1	0.21	0.85	0	39.4
Black spotted stickleback	3.25	0.90	0.90	0	NA	NA	NA	52.7

## Discussion

Selecting proper survey methods and locations is an essential step in the design of monitoring programs to inform management and conservation in coastal ecosystems around the world. Our study compared two widely used survey methods for coastal fish assemblages, underwater visual census (UVC) and beach seine netting, and assessed whether shallow nearshore assemblages commonly sampled by beach seines are comparable to those in nearby eelgrass beds often surveyed by UVC. Our results indicate that both survey methods yield comparable measures of species richness, diversity and evenness, yet species composition differed and beach seine surveys gave higher abundance estimates than UVC. Although the two methods are not directly comparable, ideally they should complement each other to assess the entire coastal fish assemblage and monitor broader changes in coastal ecosystems. Similarly, despite close proximity and considerable overlap, we found distinct differences between assemblage structure sampled in eelgrass beds and nearshore habitats. These results have important implications for how to compare already existing long-term and large-scale survey data, and how best to design appropriate future monitoring programs for coastal macrofaunal assemblages based on species of interest that can inform management of critical habitats at regional, national or international levels.

### Survey method selection

Beach seine netting and UVC are two of the most commonly used methods for surveying coastal fish or macrofauna assemblages because they are simple, fast, repeatable, cost-effective and mostly non-destructive; yet both methods have known biases that explain the results observed in our study (Brock, 1982; Piercer, Rasmussen & Leggett, 1990; Connolly, 1994; Harvey et al., 2004; Weldon et al., 2004; Ward-Paige, Flemming & Lotze, 2010; Dickens et al., 2011; Franco et al., 2012). Generally, visual surveys work well when visibility is high and for non-cryptic or sedentary species with neutral behavior toward a diver (Côté & Perrow, 2006; Murphy & Jenkins, 2010; Franco et al., 2012). However, UVC can underestimate the abundance of the most common species, with issues surrounding counting large numbers of small, mobile individuals at once, and cryptic species hiding in the seafloor or vegetation (Brock, 1982; Franco et al., 2012). This is supported by our results, both within and among estuaries, with two of the most abundant species, Atlantic silverside and mummichog, being less abundant in the UVC, and some highly cryptic species being undetected (winter flounder, flounder species) or rarely detected (smooth flounder) compared to the beach seine. Future surveys that employ the interference visual census (IVC) of cyptic fishes could address this shortcoming (Beldade & Gonçalves, 2007). Three other species not observed in the UVC were quite rare mobile fishes (gaspereau, striped bass, cunner), that may have avoided divers (Chapman & Atkinson, 1986). Finally, in turbid waters with low visibility, UVC may result in reduced observations and consequently underestimation of faunal abundance and richness, which we encountered at several study sites after heavy rainfall.

Biases discussed for the beach seine are dominated by escapees due to net snagging or rolling, fish swimming away when the net is cast, or individuals finding refuge in depressions in the seabed (Piercer, Rasmussen & Leggett, 1990; Connolly, 1994; Weldon et al., 2004; Franco et al., 2012). In our study, the beach seine surveys failed to detect two species, grubby and lady crab, which were observed in the UVC, and yielded lower abundances for rock crab, mud crab, black-spotted and 9-spine sticklebacks within or among estuary comparisons. The grubby is a mobile fish that may have avoided the seine net or left the area by the time the seine occurred. The lady crab is less mobile compared to the grubby but may have taken refuge in a depression or under a rock, escaping capture by the seine. These observations of the beach seine not detecting as many mobile species as UVC has also been found in another study by Franco et al. (2012). Overall, the beach seine found higher abundances for many species and more effectively detected rare or cryptic species (see above), similar to another study that compared survey methods (Franco et al., 2012).

Although species missed by one survey method will have influenced species composition, none were identified by SIMPER analysis as important in driving differences in assemblage composition, except for juvenile flounder, which explained 3.3% of the difference in the within-estuary comparison. In contrast, the four SIMPER species explaining >90% of the differences in the species composition (Atlantic silverside, mummichog, 4-spine stickleback, green crab) were the most common species in our surveys, with generally higher abundances in the beach seine. Thus, an underestimation of these common species by UVC may have contributed to the significant difference in species composition between methods (Brock, 1982). Lastly, since we pooled two nearshore transects to compare with one beach seine, the UVC sampled a larger overall area than the beach seine, which may have increased species richness (Soberon & Lorente, 1993). However, variability in terms of species richness between the two nearshore transects prior to pooling was low (on average ±1.66 species), and repeating the analysis with just one transect per site did not change the overall results.

In summary, despite sampling in the same location under very similar conditions, the beach seine and visual survey yielded different results in terms of total abundance and distinct differences in assemblage composition, despite considerable overlap of detected species. Thus, it is not possible to conclude which method provides a better summary of the nearshore macrofaunal assemblage, and survey selection may depend on the survey objective, the measures of interest (e.g., abundance vs. diversity), available resources (e.g., beach seine vs. divers) and environmental conditions. If the nearshore habitat is devoid of obstructions and algae or has low water visibility, the beach seine may better sample rare, cryptic and the most common species. However, beach seines can have a more destructive impact, in some cases leading to high faunal mortality (authors’ personal observation), which can be avoided with visual surveys. Whichever method is chosen, it cannot be interchanged, and needs to stay consistent over long temporal or large spatial scales to avoid sampling biases. Ideally, the two survey methods could go hand in hand, complementing each other to assess broader changes in faunal assemblages and coastal ecosystems over time and space.

### Habitat differences

Foundation species, such as seagrasses are known to increase habitat complexity, which is often positively correlated with adult faunal density, recruitment and post-settlement survival, and negatively correlated with predation risk (Preisser & Deegan, 1995; Heck, Hays & Orth, 2003; Vandermeulen, 2005; Vandermeulen, 2009; Schmidt et al., 2011; Schein et al., 2012). Thus, seagrass beds can support different macrofaunal assemblages compared to unvegetated or unstructured habitats, but there can also be considerable overlap and spill-over between adjacent habitats (Nagelkerken et al., 2001).

In our study, UVC surveys performed in shallow nearshore habitats yielded significant differences in faunal assemblage structure compared to UVC in eelgrass beds, despite their close proximity (Weldon et al., 2004). These results are consistent with other studies reporting higher abundance, richness and diversity in seagrass beds compared to unvegetated habitats (Preisser & Deegan, 1995; Hughes et al., 2002; Heck, Hays & Orth, 2003; Vandermeulen, 2005; Vandermeulen, 2009; Joseph, Locke & Godin, 2006; Schmidt et al., 2011; Schein et al., 2012). This relationship likely exists because eelgrass plants provide increased habitat complexity and structure, more ecological niches, a refuge from predation, a less turbulent environment and more abundant and diverse food sources (Preisser & Deegan, 1995; Heck, Hays & Orth, 2003; Schmidt et al., 2011). The only non-significant measure in our study was evenness, suggesting that despite significant differences in species richness and abundance, the assemblages have a similar distribution of the proportion of individuals spread over several species, with two or three abundant, and many uncommon species. However, on average evenness was higher in eelgrass beds compared to nearshore habitats, also reported in a similar study comparing seagrass beds and sandy bottom habitats in the Caribbean, which were attributed to differences in habitat complexity and productivity (Nagelkerken & Van der Velde, 2004).

In addition to summary measures of assemblage structure, species composition also significantly differed between the two habitats. Two species, green crab and grubby, were only found in nearshore habitat, although the grubby is thought to occur primarily within eelgrass beds (Lazzari, Able & Fahay, 1988). Nagelkerken et al. (2001) suggested that unvegetated habitats in close proximity to seagrass beds exhibit some species overlap due to migration and spill-over, which may explain the presence of the grubby in nearhore waters. As in the survey comparison above, the most common species contributed most to differences in species composition, namely Atlantic silverside and rock crab more abundant in eelgrass and mummichog more abundant nearshore. Similarly, Schein et al. (2012) found that eelgrass beds were dominated by Atlantic silversides, and Heck et al. (1995) found higher abundance of rock crabs in eelgrass beds compared to unvegetated habitat. In contrast, mummichogs prefer muddier habitats dominated by macroalgae (such as sea lettuce) than eelgrass (Finley et al., 2013). Thus, observed differences in the most common species and overall assemblage structure can be explained by habitat preferences and are not outweighed by spill-over or connectivity between habitats.

Given the differences in assemblage structure between nearshore and eelgrass habitats, nearshore surveys do not represent faunal assemblages in eelgrass beds. However, due to their close proximity and likelihood of spill-over from and connectivity with seagrass beds (Nagelkerken et al., 2001), nearshore assemblages are likely influenced by changes within seagrass beds. Therefore, monitoring not just one, but multiple coastal habitats, would provide the most valuable insight into overall changes in coastal ecosystems by highlighting the importance of linkages between different habitats (França, Costa & Cabral, 2009; França, Costa & Cabral, 2011; Selleslagh et al., 2009; Franco et al., 2012).

## Conclusions

In the southern Gulf of St. Lawrence, Canada, two long-term and regional-scale monitoring and research programs surveying coastal fish assemblages have been in place since 2003. Due to different survey methods and survey locations used, results are not directly comparable to each other in assessing broader changes in the entire assemblage. As new regional monitoring programs are being developed or refined around the world, such as NorSt-EMP in the Southern Gulf of St. Lawrence, multiple survey methods and habitats should ideally be combined to assess long-term and large-scale changes in estuarine and coastal ecosystems. In addition, the results of our study emphasize the ecological importance of eelgrass beds which support significantly enhanced macrofaunal abundance and diversity compared to surrounding unvegetated habitats.

## Supplemental Information

10.7717/peerj.1832/supp-1 Figure S1 Site-specific results for (A) abundance, (B) species richness, (C) diversity, and (D) evenness measured by beach seine (dark grey) and visual (white) surveys

10.7717/peerj.1832/supp-2 Figure S2 Site-specific results (mean, ±SE, *n* = 2) for (A) abundance, (B) species richness, (C) diversity, and (D) evenness measured by visual surveys in nearshore (white) and eelgrass (black) habitats

10.7717/peerj.1832/supp-3 Table S1 Names of all study sites, with latitude and longitude, date sampled, water temperature and salinity during sampling, and an indication whether they were included in the method and habitat comparisons

10.7717/peerj.1832/supp-4 Table S2 Length at maturity data for all species encountered, except for common starfish for which weight at maturity is listed and flounder sp. which were too small to identify and clearly juveniles

10.7717/peerj.1832/supp-5 Table S3 Mobile macrofauna species observed in each survey method (visual survey and beach seine) with their life stage (J, juvenile; A, adult) among five New Brunswick estuaries

10.7717/peerj.1832/supp-6 Table S4 Mobile macrofauna species observed in each habitat type (nearshore vs eelgrass bed) with their life stage (J, juvenile, A, adult) in five New Brunswick estuaries (*n* = 5)

10.7717/peerj.1832/supp-7 Supplemental Information 1 2013 New Brunswick method comparison raw data

10.7717/peerj.1832/supp-8 Supplemental Information 2 New Brunswick habitat comparison raw data

## Additional Information and Declarations

### Field Study Permissions

The following information was supplied relating to field study approvals (i.e., approving body and any reference numbers): This field research was conducted under the scientific licence to the Department of Fisheries and Oceans, # SG-RHQ-13-089.

### Data Availability

The following information was supplied regarding data availability: Survey data can be found in the Supplemental Information.
