# Supplementary material for: Comparative analysis of different survey methods for monitoring fish assemblages in coastal habitats
Source: PeerJ. 2016 Mar 21;4:e1832. doi: 10.7717/peerj.1832 (PMC4806602; doi:10.7717/peerj.1832)
Supplement: Table S1 [file peerj-04-1832-s003.docx]

evenness measured by beach seine (dark grey) and visual (white) surveys.

| Site | Latitude | Longitude | Sampling date | Temperature (°C) | Salinity (PSU) | Method | Method | Habitat |
| --- | --- | --- | --- | --- | --- | --- | --- | --- |
|  |  |  |  |  |  | within estuary | among estuaries |  |
| Cocagne 1 | 46.367240 | -64.617380 | Aug. 7, 2013 | 22.3 | 25.99 | X | X | X |
| Cocagne 3 | 46.335807 | -64.615219 | Aug. 6, 2013 | 20.5 | 22.70 | X |  |  |
| Cocagne 4 | 46.341020 | -64.573724 | Aug. 6, 2013 | 24.4 | 23.38 | X |  |  |
| Cocagne 5 | 46.331250 | -64.582930 | Aug. 6, 2013 | 22.1 | 22.57 | X |  |  |
| Cocagne 6 | 46.356470 | -64.573946 | Aug. 6, 2013 | 23.0 | 23.74 | X |  |  |
| Bouctouche | 46.499722 | -64.676484 | Aug. 8, 2013 | 23.1 | 23.98 |  | X | X |
| Kouchibouguac | 46.841667 | -64.937778 | Aug. 9, 2013 | 22.8 | 22.70 |  | X | X |
| Shippagan | 47.732050 | -64.775691 | Aug. 12, 2013 | 20.2 | 23.96 |  | X | X |
| Lamèque | 47.795556 | -64.675278 | Aug. 13, 2013 | 20.0 | 25.09 |  | X | X |

Table S1. Names of all study sites, with latitude and longitude, date sampled, water temperature and salinity during sampling, and an indication whether they were included in the method and habitat comparisons.
