# Supplementary material for: Comparative analysis of different survey methods for monitoring fish assemblages in coastal habitats
Source: PeerJ. 2016 Mar 21;4:e1832. doi: 10.7717/peerj.1832 (PMC4806602; doi:10.7717/peerj.1832)
Supplement: Table S2 [file peerj-04-1832-s004.docx]

**Table S2.** Length at maturity data for all species encountered, except for common starfish for which weight at maturity is listed and flounder sp. which were too small to identify and clearly juveniles.

| **Species** | **Length at**  **Maturity (cm)** | **Reference** |
| --- | --- | --- |
| *Alosa pseudoharengus* (Gaspereau) | 25.0 | 1 |
| *Ammodytes americanus* (American sand lance) | 12.0 | 2 |
| *Apeltes quadracus* (4-spine stickleback) | 2.5 | 3 |
| *Asterias vulgaris* (Common starfish) | 1-10 g | 4 |
| *Cancer irroratus* (Rock crab) | 2.0 | 3 |
| *Carcinus maenas* (Green crab) | 1.5 | 3 |
| *Fundulus heteroclitus* (Mummichog) | 3.0 | 3 |
| *Gasterosteus aculeatus* (3-spine stickleback) | 2.5 | 3 |
| *Gasterosteus wheatlandi* (Black spotted stickleback) | 2.5 | 3 |
| *Menidia menidia* (Atlantic silverside) | 8.0 | 3 |
| *Morone saxatilis* (Striped bass) | 12.0 | 3 |
| *Myoxocephalus aenaeus* (Grubby) | 6.2 | 2 |
| *Ovalipes ocellatus* (Lady crab) | 5.0 | 5 |
| *Panopeus sp.* (Mud crab) | 0.5 | 3 |
| *Pleuronectes putnami* (Smooth flounder) | 5.0 | 3 |
| *Pleuronectes* sp. (Flounder sp.) | NA | NA |
| *Pseudopleuronectes americanus* (Winter flounder) | 27.4 | 3 |
| *Pungitius pungitius* (9-spine stickleback) | 2.5 | 3 |
| *Tautogolabrus adspersus* (Cunner) | 5.0 | 3 |

**References for Table S2**

1. Scott WB, Scott MG. 1988. *Alosa pseudoharengus*. Atlantic Fishes of Canada 219: 105-108.
2. CAMP. 2009. Identification key. 1-20.
3. Weldon J, Garbary D, Courtenay S, Ritchie W, Godin C, Thériault M-H, Boudreau M, Lapenna A. 2004. The community aquatic monitoring project (CAMP) for measuring marine environmental health in coastal waters of the southern Gulf of St. Lawrence: 2004 overview. Canadian Technical Report in Fisheries and Aquatic Sciences 2624: viii+53 p.
4. Menge BA. 1986. A preliminary study of the reproductive ecology of the seastars *Asterias vulgaris* and *A. forbesi* in New England. Bulletin of Marine Science 39(2): 467-476.
5. Stehlik L, Pikanowski R, McMillan D. 2004. The Hudson-Raritan Estuary as a crossroads for distribution of blue (*Callinectes sapidus*), lady (*Ovalipes ocellatus*), and Atlantic rock (*Cancer irroratus*) crabs. Fishery Bulletin 102(4): 693-710.
