# Supplementary material for: Comparative analysis of different survey methods for monitoring fish assemblages in coastal habitats
Source: PeerJ. 2016 Mar 21;4:e1832. doi: 10.7717/peerj.1832 (PMC4806602; doi:10.7717/peerj.1832)
Supplement: Table S3 [file peerj-04-1832-s005.docx]

**Table S3.**  Mobile macrofauna species observed in each survey method (visual survey and beach seine) with their life stage (J = juvenile, A = adult) among five New Brunswick estuaries.

| **Species** | **Visual**  **Survey** | **Beach**  **Seine** |
| --- | --- | --- |
| *Alosa pseudoharengus* (Gaspereau) |  | J |
| *Ammodytes americanus* (American sand lance) | J | A |
| *Apeltes quadracus* (4-spine stickleback) | J/A | J/A |
| *Cancer irroratus* (Rock crab) | A | A |
| *Carcinus maenas* (Green crab) | A | J/A |
| *Fundulus heteroclitus* (Mummichog) | A | J/A |
| *Gasterosteus aculeatus* (3-spine stickleback) | J | A |
| *Gasterosteus wheatlandi* (Black spotted stickleback) | J | A |
| *Menidia menidia* (Atlantic silverside) | J/A | J/A |
| *Morone saxatilis* (Striped bass) |  | J |
| *Myoxocephalus aenaeus* (Grubby) | J/A |  |
| *Ovalipes ocellatus* (Lady crab) | A |  |
| *Panopeus* sp. (Mud crab) | A | A |
| *Pleuronectes putnami* (Smooth flounder) | J/A | J/A |
| *Pleuronectes* sp. (Flounder sp.) |  | J |
| *Pseudopleuronectes americanus* (Winter flounder) |  | J/A |
| *Pungitius pungitius* (9-spine stickleback) | J/A | A |
| *Tautogolabrus adspersus* (Cunner) |  | J |
|  |  |  |
| Total (18) | 13 | 16 |
