# Supplementary material for: Comparative analysis of different survey methods for monitoring fish assemblages in coastal habitats
Source: PeerJ. 2016 Mar 21;4:e1832. doi: 10.7717/peerj.1832 (PMC4806602; doi:10.7717/peerj.1832)
Supplement: Table S4 [file peerj-04-1832-s006.docx]

**Table S4**. Mobile macrofauna species observed in each habitat type (nearshore vs eelgrass bed) with their life stage (J = juvenile, A = adult) in five New Brunswick estuaries (n=5).

| **Species** | **Nearshore** | **Eelgrass**  **Bed** |
| --- | --- | --- |
| *Alosa pseudoharengus* (Gaspereau) |  | J |
| *Apeltes quadracus* (4-spine stickleback) | J/A | J/A |
| *Asterias vulgaris* (Common starfish) |  | A |
| *Cancer irroratus* (Rock crab) | A | A |
| *Carcinus maenas* (Green crab) | A |  |
| *Fundulus heteroclitus* (Mummichog) | A | J/A |
| *Gasterosteus wheatlandi* (Black spotted stickleback) |  | A |
| *Menidia menidia* (Atlantic silverside) | J/A | J/A |
| *Myoxocephalus aenaeus* (Grubby) | J/A |  |
| *Ovalipes ocellatus* (Lady crab) | A | A |
| *Panopeus* sp. (Mud crab) | A | A |
| *Pleuronectes* sp. (Flounder sp.) | J | J/A |
| *Pungitius pungitius* (9-spine stickleback) | J/A | J/A |
|  |  |  |
| Total (13) | 10 | 11 |
